# Supplementary material for: The ldp1 Mutation Affects the Expression of Auxin-Related Genes and Enhances SAM Size in Rice
Source: Plants (Basel). 2024 Mar 7;13(6):759. doi: 10.3390/plants13060759 (PMC10975181; doi:10.3390/plants13060759)
Supplement: Supplementary file 1 [file plants-13-00759-s001.zip › Figures and Tables description of the supporting materials.pdf]

Table S1. Primers used in this study

Table S2. Sequencing Sample qualities for RNA-seq

Table S3. Genetic phenotype analysis of *ldp1*

Table S4. Gene annotations in the fine mapping interval

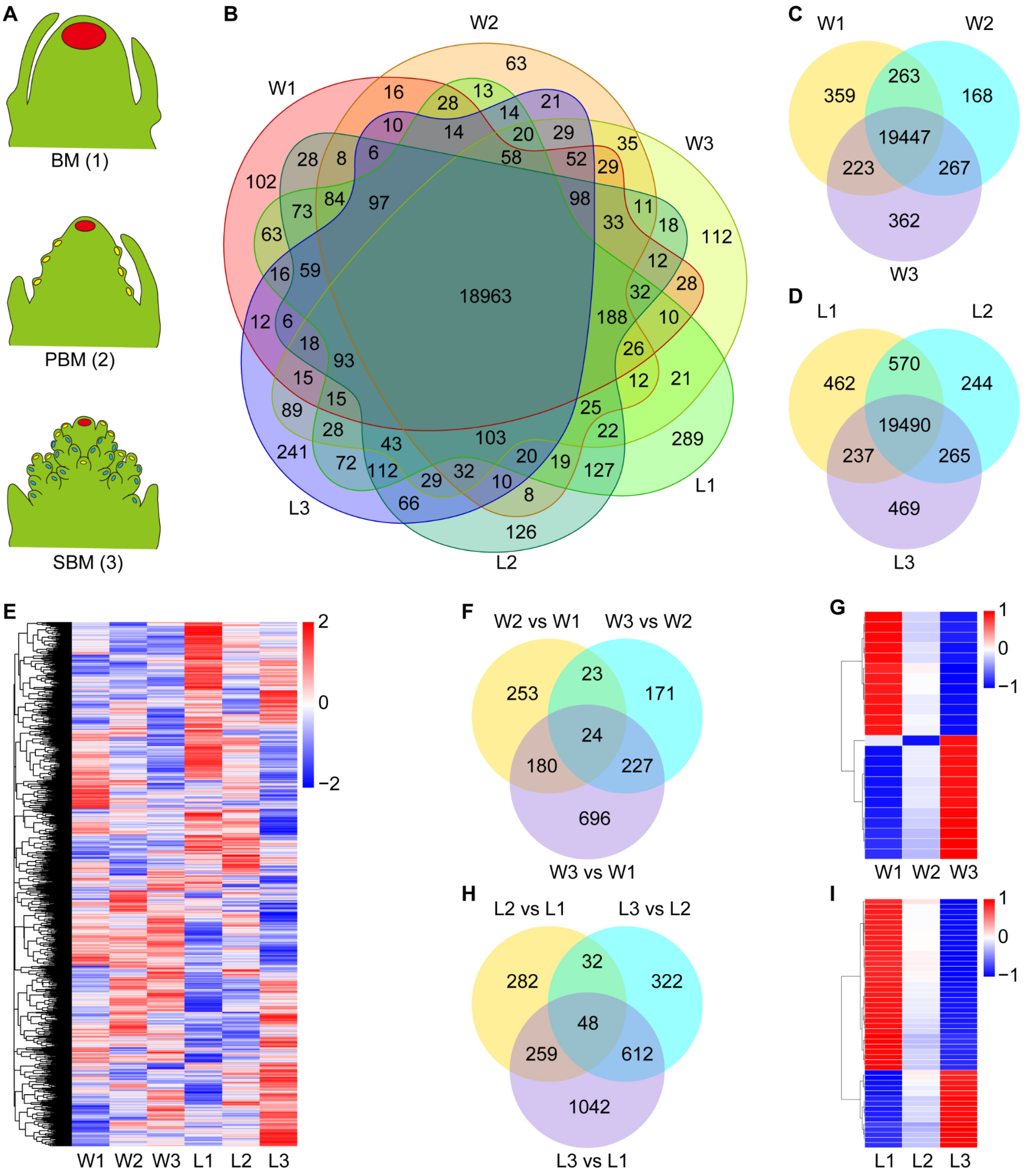

Figure S1. Gene expression analysis during the three developmental stages of young panicle between WYJ7 and ldp1 rice. (A) Cartoon diagram depicting three developmental stages of

young inflorescence differentiation; (B) Venn diagram of gene expression in six tissues; (C) Venn diagram of gene expression during the three developmental stages of young panicle in WYJ7; (D) Venn diagram of gene expression during the three developmental stages of young panicle in *ldp1*; (E) Expression features cluster analysis of the expressed genes in six tissues; (F) Venn diagram displaying the number of DEGs in each comparisons in WYJ7; (G) Expression features cluster analysis of the shared 24 DEGs in WYJ7; (H) Venn diagram displaying the number of DEGs in each comparisons in *ldp1*; (I) Expression features cluster analysis of the shared 24 DEGs in *ldp1*. The clustering method adopts hierarchical clustering, horizontally clustering genes with similar expression patterns (FPKM values close) horizontally, and normalizes the rows using Z-score; BM, bract meristem; PBM, primary branch meristem; SBM, secondary branch meristem. The stages W1-3 and L1-3 indicate the three developmental stages of BM, PBM, and SBM stages in in WYJ7 and *ldp1*, respectively.

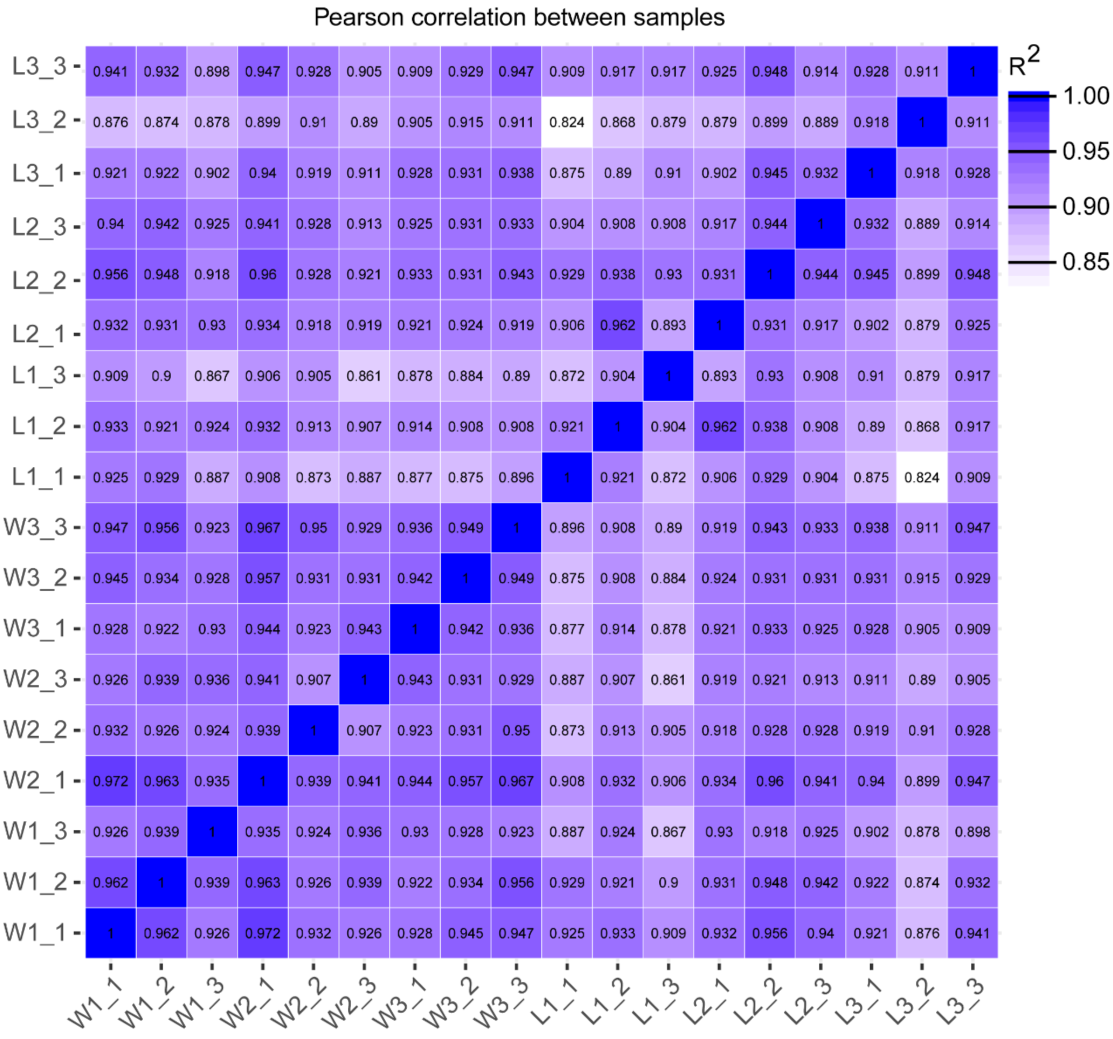

Figure S2. Pearson correlation coefficient of sequencing samples. W1-3 and L1-3 are as described in Figure S1. The suffix _1-3 in these labels represent the three independent biological replicates.

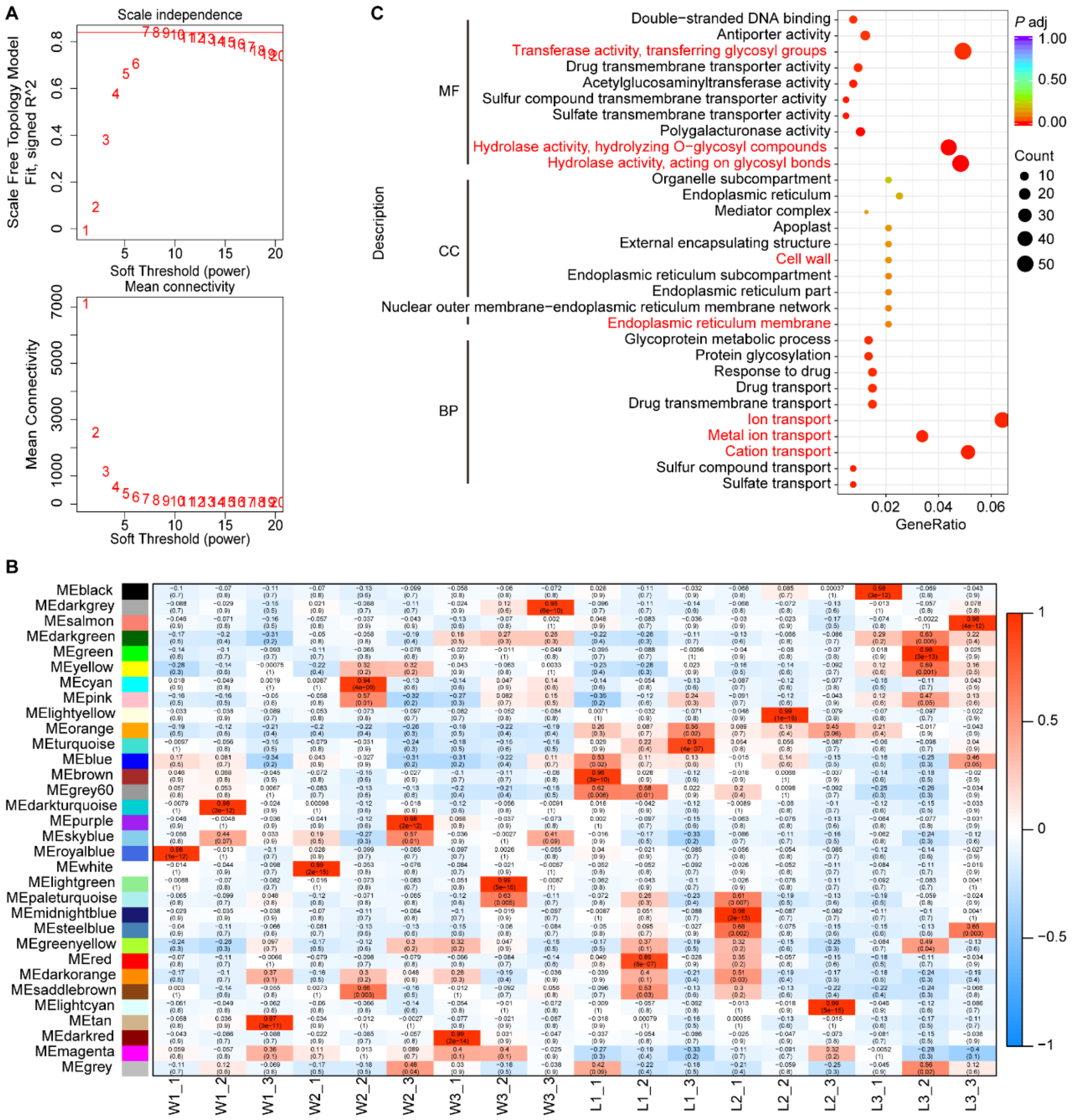

Figure S3. WGCNA analysis. (A) Weight parameter β for gene network construction; (B) Heatmap of module-sample correlation; (C) GO functional enrichment of genes in the Turquoise module, with red annotations representing the term with the most enrichment in biological process (BP), cellular component (CC), and molecular function (MF), respectively. W1-3 and L1-3 are as described in Figure S1. The suffix _1-3 are as described in Figure S2.

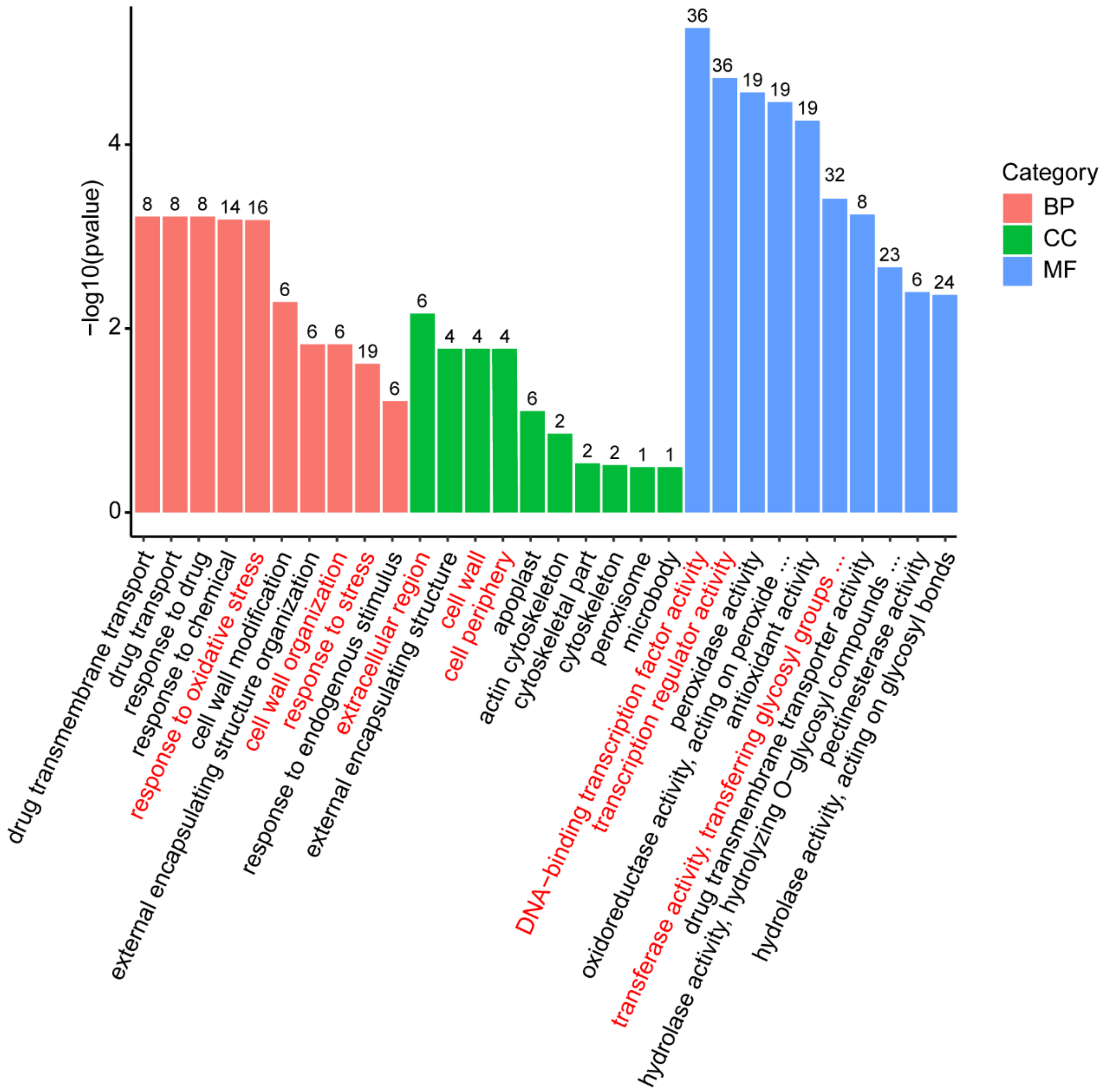

Figure S4. GO functional enrichment analysis of differentially expressed genes between L1 and W1 at the first bract differentiation stage. Red annotations are as described in Figure S3.

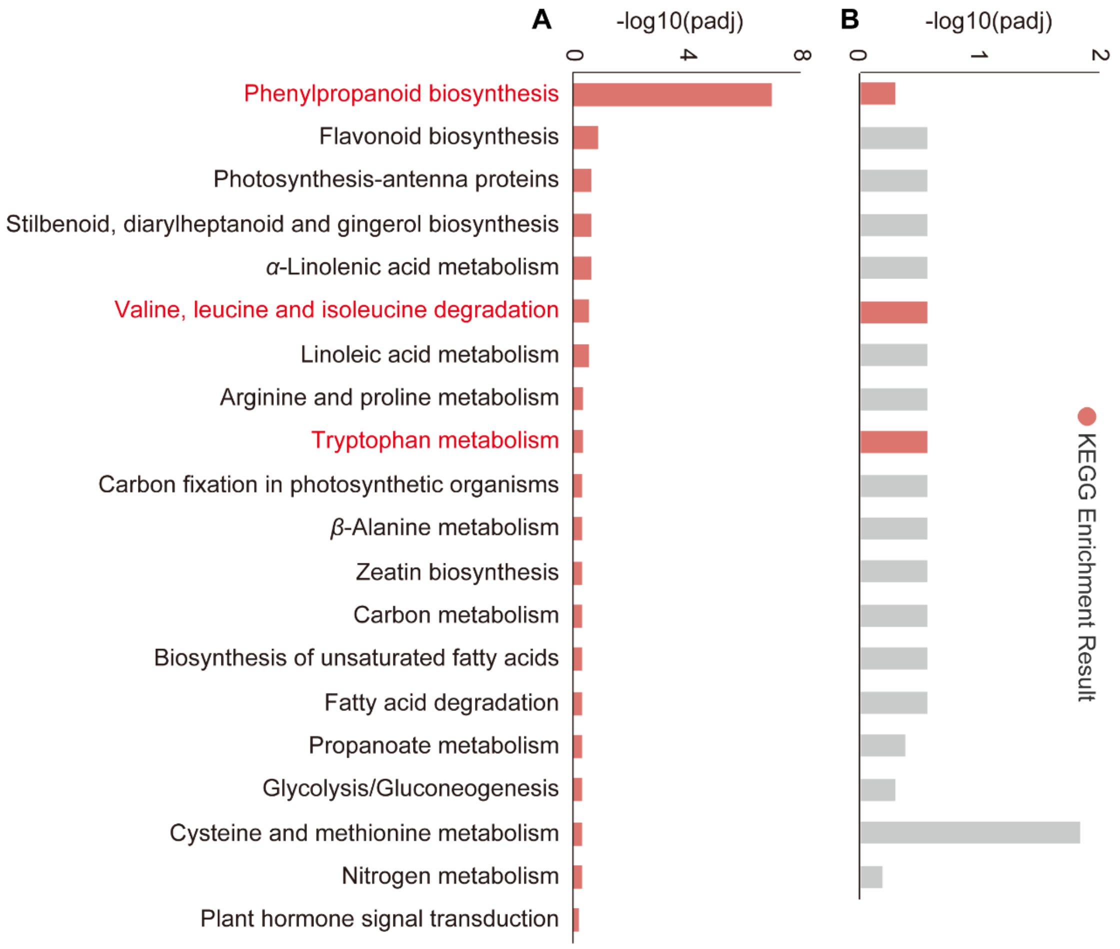

Figure S5. Comparison of KEGG enrichment maps for DEGs. (A) Functional classifications of DEGs in the comparison between L1 and W1 during the BM stage; (B) Functional classifications of 83 shared DEGs across the three developmental stages of young panicle. Red annotations representing shared enrichment in tryptophan metabolism and valine, leucine, and isoleucine biosynthesis pathways. W1 and L1 indicate the BM stage in WYJ7 and *ldp1*, respectively.
