# Supplementary figures and images for: The ldp1 Mutation Affects the Expression of Auxin-Related Genes and Enhances SAM Size in Rice
Source: Plants (Basel). 2024 Mar 7;13(6):759. doi: 10.3390/plants13060759 (PMC10975181; doi:10.3390/plants13060759)

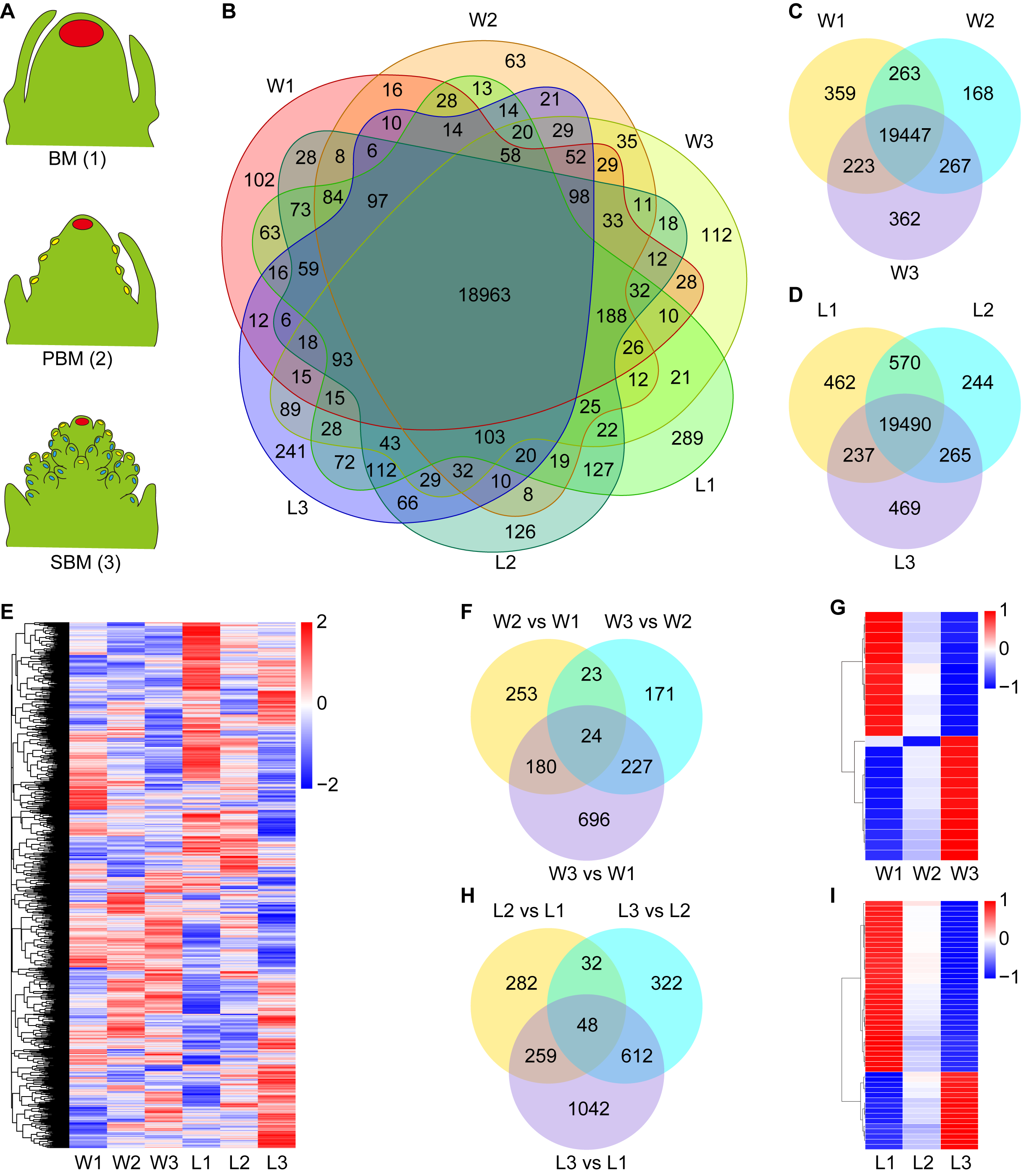

Supplement: Supplementary file 1 [file plants-13-00759-s001.zip › Figure S1 .tif]

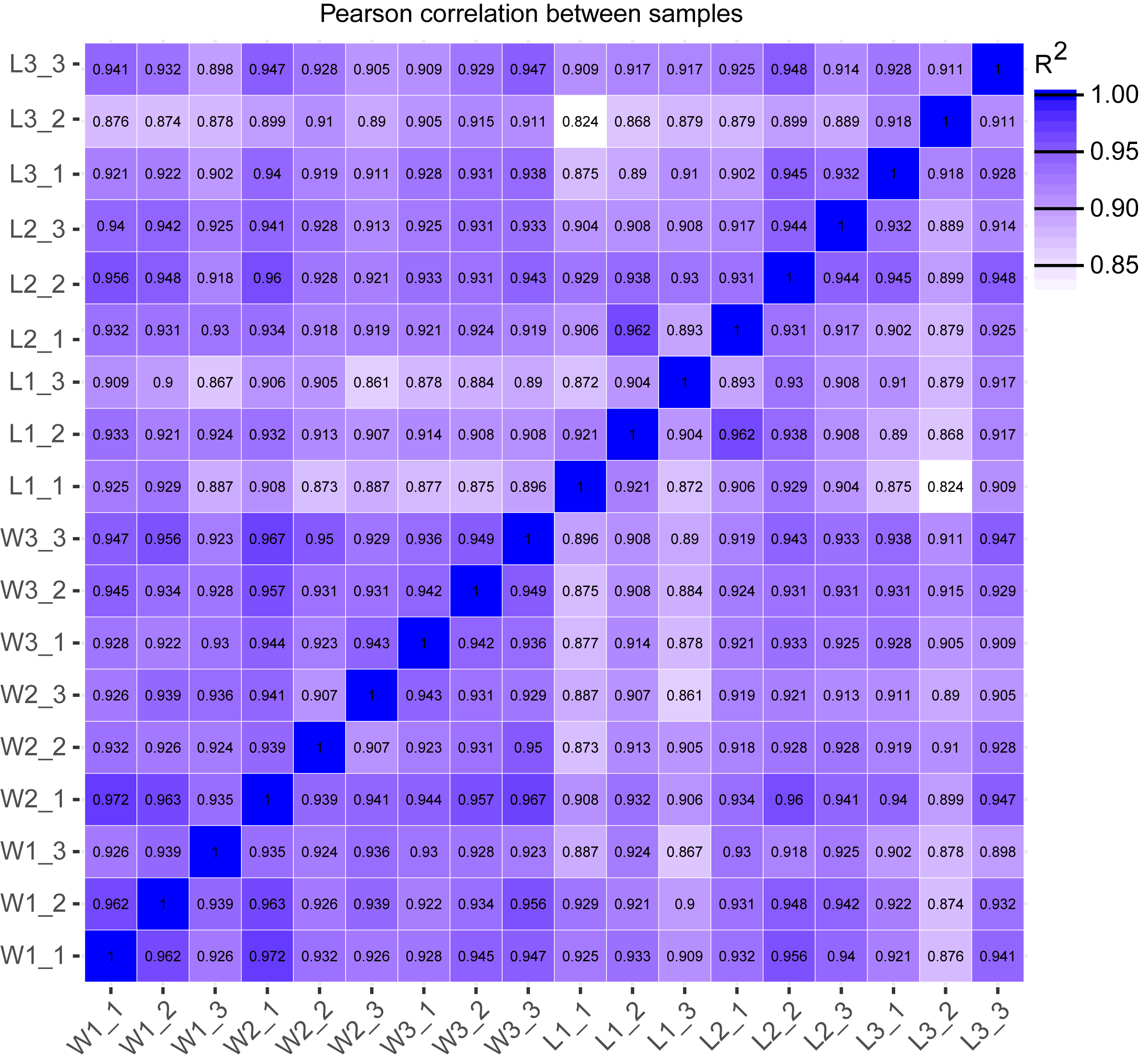

Supplement: Supplementary file 1 [file plants-13-00759-s001.zip › Figure S2.tif]

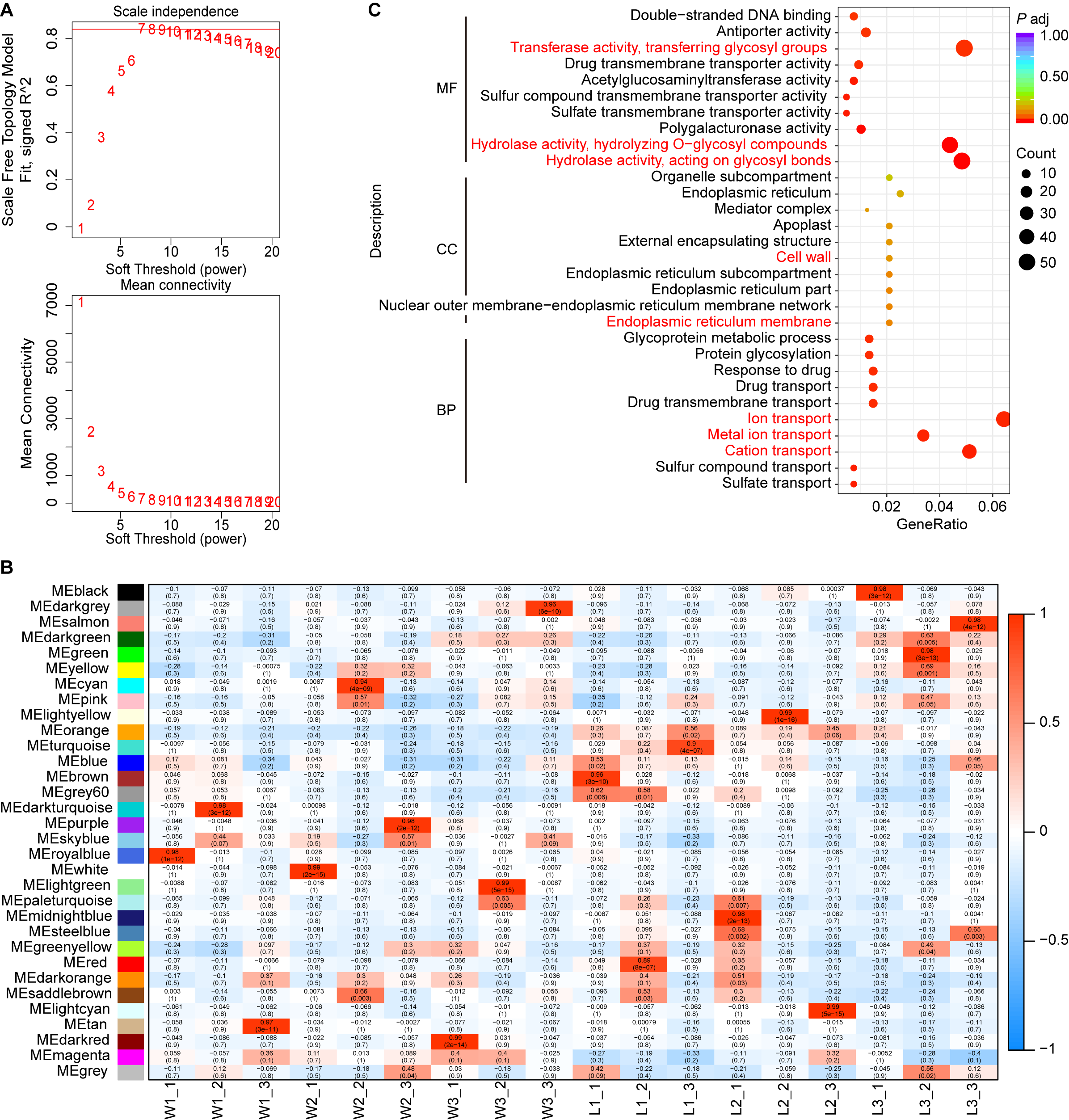

Supplement: Supplementary file 1 [file plants-13-00759-s001.zip › Figure S3.tif]

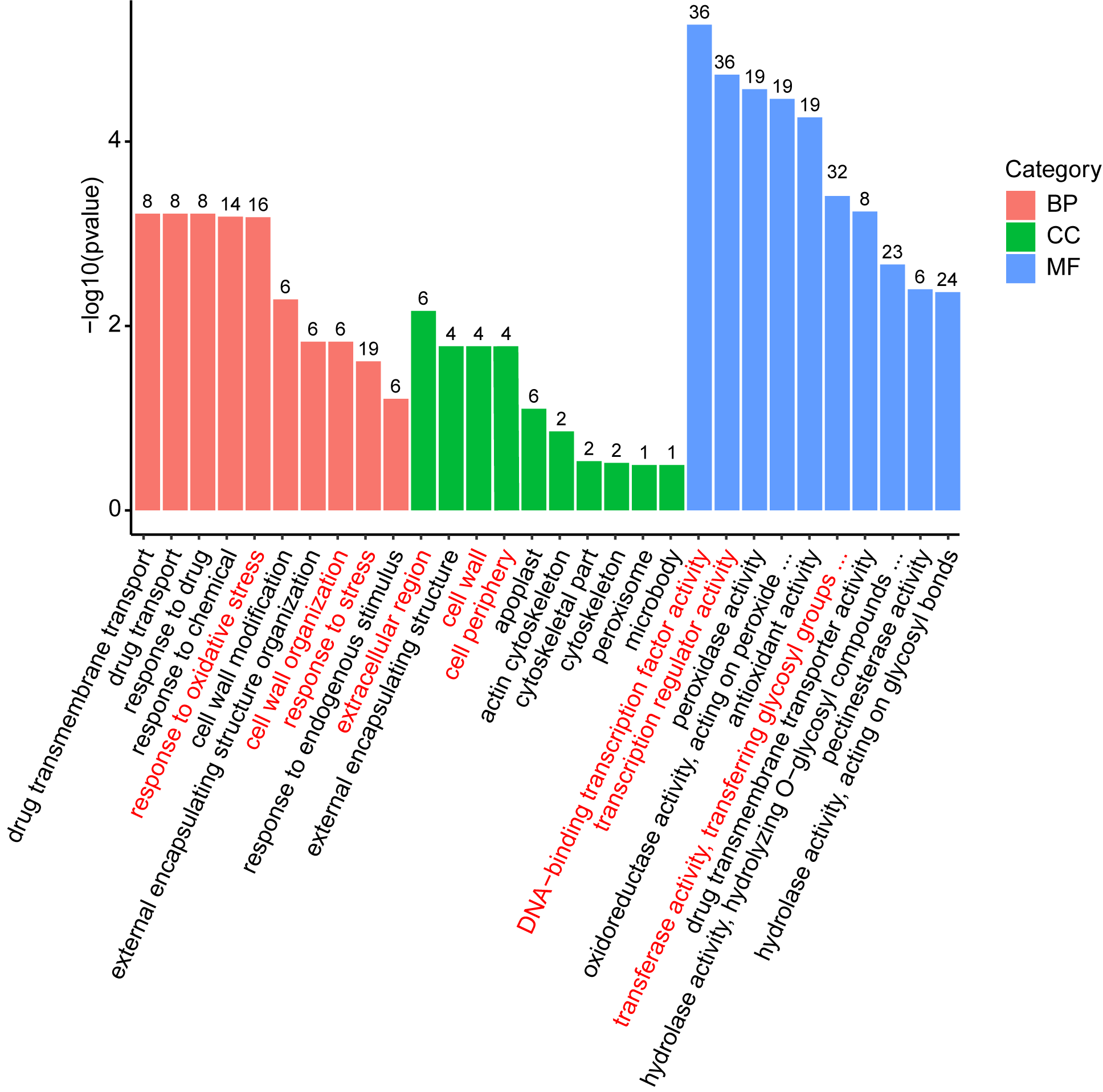

Supplement: Supplementary file 1 [file plants-13-00759-s001.zip › Figure S4.tif]

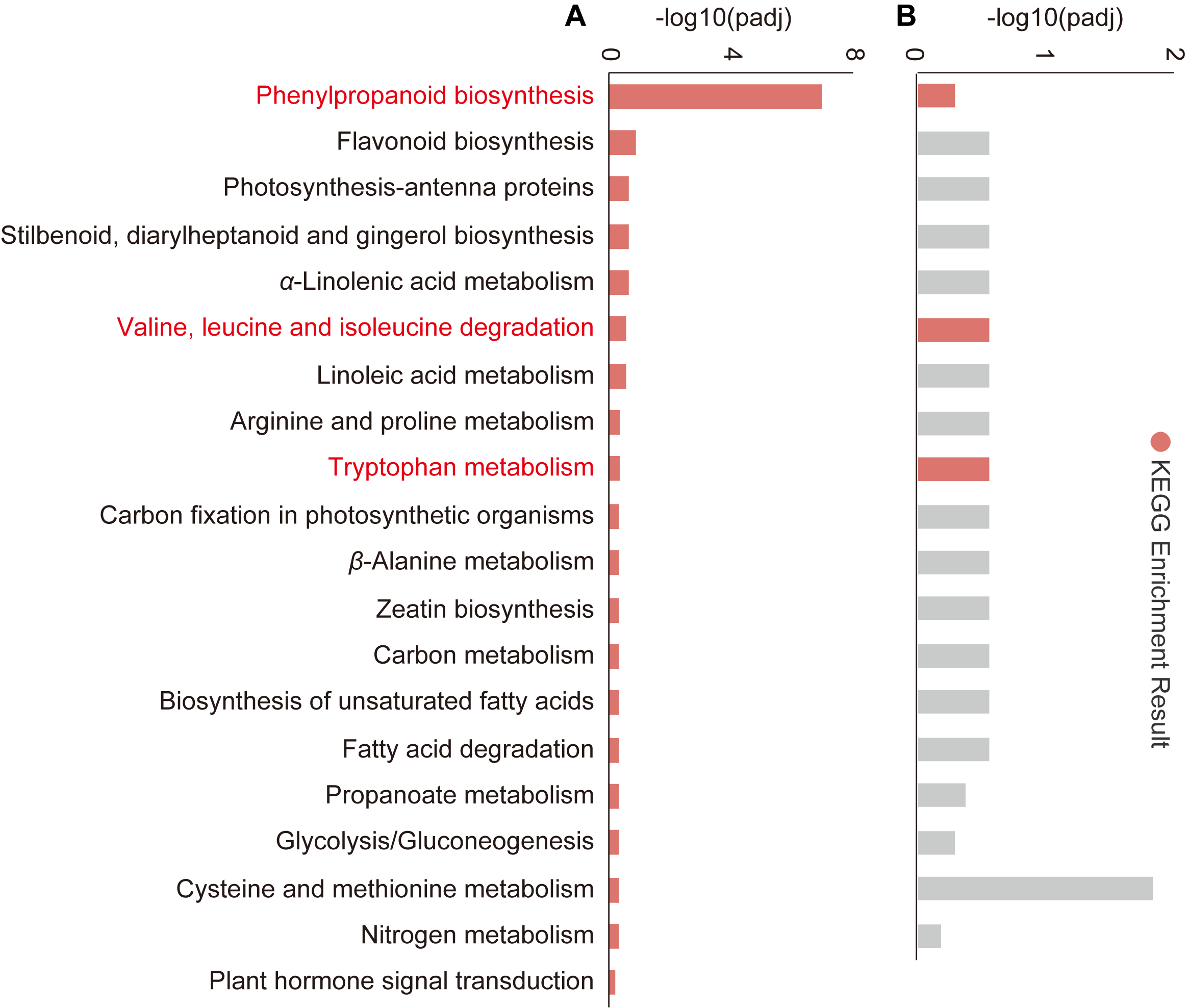

Supplement: Supplementary file 1 [file plants-13-00759-s001.zip › Figure S5.tif]
